# Three Lectures Preliminary to a Course on the Principles and Practice of Surgery

**Published:** 1850-03

**Authors:** 


*Three Lectures Preliminary to a Course on the Principles and Practice of Surgery. Delivered on the 4th, 8th and 9th of Oct.,* 1849, *before the Medical Class of the University of Pennsylvania.* By William Gibson, M. D., LL. D., Professor of Surgery, &c. Published by the Class. Philadelphia: 1850. pp. 57.

The season for valedictories from our medical professors is already nigh at hand, yet we have still to regret the want of opportunity to return acknowledgments for the many interesting introductories with copies of which we have been favored.

Thankful we sincerely are for each of these annual offerings from the professorial rostra; and we would be unfeignedly glad to vary and enrich our pages with many portions of their polished treasures, could we do so without trenching on the rights of more urgent, although not more tempting, claimants. As it is, we have surrendered, for the nonce, to Dr. Gibson's three "preliminary" lectures; not so much on account of the triple debt of gratitude their numerical excess has put us under, as for the refreshing entertainment their perusal has afforded us in the midst of melancholy heaps of drier food for thought.

They belong to a class of introductory discourses which must always find a cordial welcome amongst all hearers and readers of whatever predilections, and one which we of the company of official lookers-on have many reasons for regarding with peculiar satisfaction.

It is neither the information they convey, nor the agreeable form in which their matter is presented, that renders essays of this kind so eminently attractive in our view; nor is it the guaranty they suggest of a desire on the part of their enterprising authors, to master, at the fountain head and for the purposes of teaching, the latest improvements in their respective branches. Strong as these recommendations manifestly are, and great as we feel the charm must be to him who can revel *haud inexpertus* in the thousand glorious recollections roused by the graphic history of scenes and persons once familiar to his daily sight—still we attach far higher value to them for the sake of the powerful stimulus such stirring pictures inevitably give to the pursuit of knowledge in the minds of the embryo philosophers to whom they are especially addressed. Nowhere can the ambitious student meet with a source of impulse that would urge him so irresistibly to seek the means of future advancement in a career of European observation and comparison; and nowhere could he find a more effectual inducement to emulate in his own land the most exalted efforts of the great intellects of the older and more crowded world abroad.

But these occasional remarks are fast making away with the space allotted for the special notice of the pamphlet which has given rise to them. A very brief and cursory sketch is all that need be given here of its particular contents; and we beg leave to refer to the brochure itself for a better acquaintance with its scope and character.

Suffice it to say, that the professor's object was to present, through his customary medium of introductories, a continuation of his portraiture of the celebrated medical men and institutions of England and the continent of Europe, noted by him in the course of his last tour abroad. The three lectures now before us are chiefly occupied with an account of the "prominent medical men and schools of Germany."

Beginning with a lively *coup d'œil* of Aix La Chapelle, he takes us most agreeably through Bonn and up the Rhine to Wiesbaden and Heidelberg. Sojourning at the last old town, we are favored with a life-like view of its Tiedemann, his museum, his works, his homes, his family, and his colleagues, Chelius, Henle, Nægele and others—and thus endeth lecture first.

The second lecture is devoted, first to Liebig and his fellows, Bischoff, Wehren and others at Giessen, and then to Müller, Dieffenbach, and some of their associates and doings and places of resort at Berlin.

Lecture third carries us out of Germany into the land of dykes, and canals, and inimitable pictures, since we are introduced by way of Hamburg and the Zuyder Zee to Amsterdam with its worthies, its lions and its works of art. Next we have Leyden, with its famous university and anatomical collections; then Harlem and the Hague, with its unrivalled gallery of Dutch and Flemish paintings. Thence to Rotterdam, and out of Holland to Antwerp, Brussels and Paris. Here, we are told, our author took up his "abode again for several weeks, living the greater part of the time in the hospitals and in the society of the most distinguished professional men. Of these," continues he, "I had fondly hoped, before this, to have given a full account. But alas! owing to constant and overwhelming engagements with lectures and professional duties, I have been unable to fulfil, entirely, the promise made to a former class. To make amends for this delinquency, I propose, *Deo volente*, to print the ensuing summer a new edition of my Rambles in Europe in 1839, and to add to it a full account of my late pilgrimage in 1847—trusting that both, thus brought forward in the shape of a volume, may not only prove instructive and agreeable to students and practitioners throughout our country, but serviceable, as a guide, to those who may be willing and able hereafter to visit the scenes from which I myself have derived so many advantages and so much satisfaction. In the meantime, 'Vive vale que.'" We echo the wish. May he live a thousand years, and may the shadow of his introductories never be less!

